# Inflammatory Consequences of Maternal Diabetes on the Offspring Brain: a Hippocampal Organotypic Culture Study

**DOI:** 10.1007/s12640-019-00070-6

**Published:** 2019-06-13

**Authors:** Katarzyna Głombik, Ewa Trojan, Anna Kurek, Bogusława Budziszewska, Agnieszka Basta-Kaim

**Affiliations:** Department of Experimental Neuroendocrinology, Maj Institute of Pharmacology, Polish Academy of Sciences, 12 Smętna St, 31-343 Kraków, Poland

**Keywords:** Maternal diabetes, Hippocampus, Inflammation, Antidiabetic drugs

## Abstract

Gestational diabetes is a disorder associated with abnormal chronic inflammation that poses a risk to the developing fetus. We investigated the effects of experimentally induced diabetes (streptozotocin model) in Wistar female rats on the inflammatory status of the hippocampi of their offspring. Additionally, the impact of antidiabetic drugs (metformin and glyburide) on inflammatory processes was evaluated. Organotypic hippocampal cultures (OHCs) were prepared from the brains of the 7-day-old rat offspring of control and diabetic mother rats. On the 7th day in vitro, the cultures were pretreated with metformin (3 μM) or glyburide (1 μM) and then stimulated for 24 h with lipopolysaccharide (LPS, 1 μg/ml). The OHCs obtained from the offspring of diabetic mothers were characterized by the increased mortality of cells and an enhanced susceptibility to damage caused by LPS. Although we showed that LPS stimulated the secretion of pro-inflammatory cytokines (IL-1β, IL-6, TNF-α) in the control and diabetic cultures, the levels of IL-1β and IL-6 in the OHC medium obtained from the offspring of diabetic mothers were more pronounced. In the diabetic cultures, enhanced levels of TLR-4 and the overactivation of the NLRP3 inflammasome were demonstrated. Metformin and glyburide pretreatment normalized the LPS-induced IL-1β secretion in the control and diabetic cultures. Furthermore, glyburide diminished both: LPS-induced IL-6 and TNF-α secretion in the control and diabetic cultures and increased NF-κB p65 subunit phosphorylation. Glyburide also diminished the levels of the NLRP3 subunit and caspase-1, but only in the diabetic cultures. The results showed that maternal diabetes affected inflammatory processes in the offspring brain and increased hippocampal sensitivity to the LPS-induced inflammatory response. The use of antidiabetic agents, especially glyburide, had a beneficial impact on the changes caused by maternal diabetes.

## Introduction

Diabetes (diabetes mellitus) is a heterogeneous group of disorders characterized by hyperglycemia that results from a complete or relative insufficiency of insulin secretion and/or insulin action (Tripathi and Srivastava 2006). Among the many types of diabetes, the gestational form seems particularly dangerous. Many epidemiological studies have demonstrated that diabetes during pregnancy poses risks not only to the mother but also to her child because the developing fetal tissues are exceptionally sensitive to metabolic disturbances in the mother’s body (Johns et al. 2018). It has been indicated that fetal exposure to maternal diabetes increases the risk of obesity and glucose intolerance (Kim et al. 2012). There are also strong arguments showing that the risk of diabetes in adulthood is more prevalent among subjects who were exposed to maternal diabetes in utero. The role of maternal inheritance in diabetes has been reported in many studies (Portha et al. 2011; Armengaud et al. 2018). It has been shown that even the newborns of mothers suffering from pharmacologically controlled diabetes are at risk of various complications, including cardiomyopathies, organic heart disease, neural tube anomalies, atelencephaly, and metabolic disturbances manifested by hypoglycemia and other metabolic symptoms in the central nervous system (Hornberger 2006; Salbaum and Kappen 2010; Zhao and Reece 2013). For this reason, it is thought that diabetes during pregnancy may be a harmful factor leading to malfunction in the developing brain of offspring because of metabolic abnormalities and perinatal complications (Georgieff 2006; Babiker et al. 2015) and may result in alterations in neurogenesis, migration, differentiation, and cell survival (Carrapato and Marcelino 2001; Cederberg et al. 2003) as well as excessive inflammatory changes (Vuong et al. 2017). Several studies have highlighted that, in the brain, inflammatory processes induced by diabetes are involved in brain dysregulation. Data from different experimental models have indicated that neuronal dysfunction is associated with the prolonged activation of NF-κB and the expression of pro-inflammatory cytokines (Muriach et al. 2014). NF-κB activation plays a key role in the pathobiology of diabetes and its complications, including in the brain. Other studies have shown that pro-inflammatory cytokines (e.g., IL-6 and TNF-α) are involved in brain dysfunction caused by diabetes (Patel and Santani 2009). Recently, large protein complexes called inflammasomes, primarily NLRP3, have been proposed to play an important role in inflammatory brain processes caused by high glucose levels (Ward and Ergul 2016). NLRP3 is a large multiprotein cytoplasmic complex (> 700 kDa) composed of NLRP3 subunit, an adaptor protein named apoptosis-associated speck-like protein containing a CARD (ASC) and pro-caspase-1. The activation of NLRP3 leads to the oligomerization and recruitment of ASC. NLRP3 includes an N-terminal pyrin domain (PYD), which physically interacts with the PYD domain of ASC and thus facilitates the subsequent recruitment and activation of pro-caspase-1. Then, caspase-1 is autocatalytically cleaved to produce its active form and is responsible for the maturation of pro-IL-1β and pro-IL-18 into their biologically active forms (Lamkanfi and Kanneganti 2010; Maldonado-Ruiz et al. 2017). Furthermore, it is known that harmful factors during pregnancy lead to the activation of many other inflammatory processes in the brain. It has been shown that gestational diabetes (induced by diet) stimulates the activation of microglia and induces chronic neuroinflammation, which is manifested by the increased secretion of pro-inflammatory factors, such as IL-2, IFN-γ, and the chemokine MCP-1, in the offspring brain (Vuong et al. 2017; Edlow 2017). Based on these data, we suggest that maternal diabetes can be a risk factor for exaggerated neuroinflammation in the offspring brain. Therefore, the aim of our study was to determine the effect of experimentally induced diabetes in dams on inflammatory activation in the brains of their offspring. Diabetes induced experimentally through treatment with β-cytotoxic agents, such as streptozotocin (STZ), is well described. STZ is an alkylating compound that causes DNA alkylation and increases free radical and nitric oxide production, thus leading to DNA damage and cytotoxicity (Kiss et al. 2009). STZ-induced diabetes is caused by the selective destruction of insulin-secreting pancreatic β-cells; therefore, STZ is the first choice for diabetes induction in animal models (King 2012; Furman 2015). Standard protocols of STZ injections at different doses of the compound can be used to reproduce gestational diabetes in animals when injected during the neonatal period, before mating or during pregnancy. These models are very useful in basic studies that may translate into fetal outcomes in pregnant women presenting with uncontrolled clinical diabetes (Kiss et al. 2009).

The present study was carried out in hippocampal organotypic culture, which is an innovative and potent in vitro model that permits several cell types of the brain to be studied in a complex network. Hippocampal cultures retain cell architecture and connections, as well as cell-to-cell functional interactions (Sun et al. 2010). Furthermore, this in vitro model offers the possibility of easy pharmacological manipulations for investigating the mechanisms of inflammatory processes in the brain. Therefore, we also evaluated the effect of antidiabetic drugs (metformin and glyburide) on inflammatory processes, specifically on cytokine levels in organotypic cultures of the hippocampi from the 7-day-old offspring of control and diabetic dams.

Metformin, an oral glucose-lowering agent, is still the gold standard in the treatment of diabetes (Marshall 2017). The blood glucose-lowering effect of metformin is caused by its inhibitory effect on hepatic glucose production and by peripheral tissue sensitization to insulin action. From the clinical point of view, it is important that metformin treatment does not generate hypoglycemia but augments glucose uptake in the periphery, reduces the requirement for insulin and is characterized by a positive influence on the cardiovascular system and lipid metabolism (Wróbel et al. 2017). In addition to the glucose-lowering action of metformin, the anti-inflammatory and immunosuppressive properties of metformin (Saisho 2015) have been demonstrated in many studies conducted on peripheral and central inflammation (Jin et al. 2014; Vigili de Kreutzenberg et al. 2015; Jiang et al. 2017). The second drug used in the study, glyburide (also known as glibenclamide), is a widely used second-generation sulfonylurea. According to DrugBank, glyburide is effective in lowering blood glucose in noninsulin-dependent diabetic patients whose hyperglycemia cannot be satisfactorily controlled by diet alone. Glyburide acts by inhibiting ATP-sensitive K+ (KATP) channels in pancreatic β cells (Ashcroft 2005) and by effectively inhibiting the NLRP3 inflammasome. In STZ-induced diabetic rats, glyburide treatment diminishes oxidative stress and inflammation in the periphery (Alotaibi et al. 2018) and prevents the inflammation and fibrosis of the atria (Hayami et al. 2016).

## Materials and Methods

### Animals

Wistar rats (200–250 g) were purchased from Charles River Corporation (Germany). Rats were kept in an animal housing facility at a room temperature of 22 ± 1 °C and with a 12/12 h light/dark cycle (lights on at 07.00 a.m.) with food and water available *ad libitum*.

### Induction of Diabetes

To induce diabetes, adult females were injected intraperitoneally (i.p.) with streptozotocin (STZ). One week after diabetes induction, females were mated with syngenic non-diabetic males of the same strain. Blood glucose concentration (BGC) in animals was measured using a commercially available digital glucometer (Abbot Optimum Xido, UK). Only STZ-treated females exhibiting BGC > 350 mg/dL on the day of mating and on delivery day were used in the study (Hami et al. 2013).

### Establishment of Organotypic Cultures

Seven-day-old male pups from both control and diabetic dams were decapitated with scissors, and their brains were quickly and aseptically removed and placed in an ice-cold working buffer (96% HBSS; 3.5% glucose; 0.3% penicillin and streptomycin; and HEPES (to maintain the pH); all reagents were obtained from Gibco, UK). The hippocampi were separated, transferred to Teflon discs, and cut into 350μm slices using a McIlwain tissue chopper. Organotypic hippocampal cultures were established according to the method described by Stoppini (Stoppini et al. 1991) with slight modifications. Cultures were transposed to Millicell-CM (Merck-Millipore, USA) membranes for further growth. In 6-well plates, the membranes were pre-equilibrated with 1 ml of culture medium (containing 50% DMEM + GlutaMax™-I, pH 7.4; 20.5% HBSS; 25% horse serum; 0.1 mg/ml glucose; 1% amphotericin B, 0.4% penicillin and streptomycin; 1% B-27 supplement; and HEPES (to maintain the pH); all reagents were obtained from Gibco, UK). The cultures were maintained for 7 days under standard conditions in an incubator (37 °C) with an adjustable CO_2_ flow (5%). The cultures were initiated in regular medium containing 25% horse serum, which was then gradually (from DIV 4th to 7th) tapered down to a serum-free medium (containing 50% DMEM F-12, pH 7.4; 44% HBSS; 0.1 mg/ml glucose; 1% amphotericin B; 0.4% penicillin and streptomycin, 1% B-27, 1% N-2; and HEPES (to maintain the pH) all reagents were obtained from Gibco, UK). The medium was first changed 24 h after culture establishment (half of the volume (0.5 ml)) and then every 48 h (whole volume (1 ml)). On the 7th day in vitro medium was changed to a serum-free medium. The hippocampal cultures were pretreated for 30 min with metformin or glyburide and then stimulated with bacterial endotoxin.

### Chemicals and Drugs

The following agents and drugs were used: streptozotocin (STZ), lipopolysaccharide (LPS), metformin (MET), and glyburide (GLI). All chemicals were obtained from Sigma-Aldrich, USA.

In vivo experiment: Streptozotocin (STZ) was dissolved in freshly prepared 0.1 M citrate buffer (Sigma Aldrich, USA). Adult females were injected once (i.p.) with STZ at a dose of 45 mg/kg body weight. Control animals were injected with vehicle (i.p.).

In vitro experiment: On the 7th day, the hippocampal cultures were pretreated for 30 min with metformin (MET, final concentration in the well: 3 μM) or glyburide (GLI, final concentration in the well: 1 μM) at doses that effectively inhibit LPS-induced LDH and NO release. The antidiabetic drugs were dissolved in accordance with the supplier’s recommendations. Next, the cultures were stimulated by adding bacterial endotoxin (lipopolysaccharide (LPS); final concentration in the well: 1 μg/ml, 0111:B4) to the medium for 24 h. Control cultures were treated with the appropriate vehicle.

**Fig. 1:**
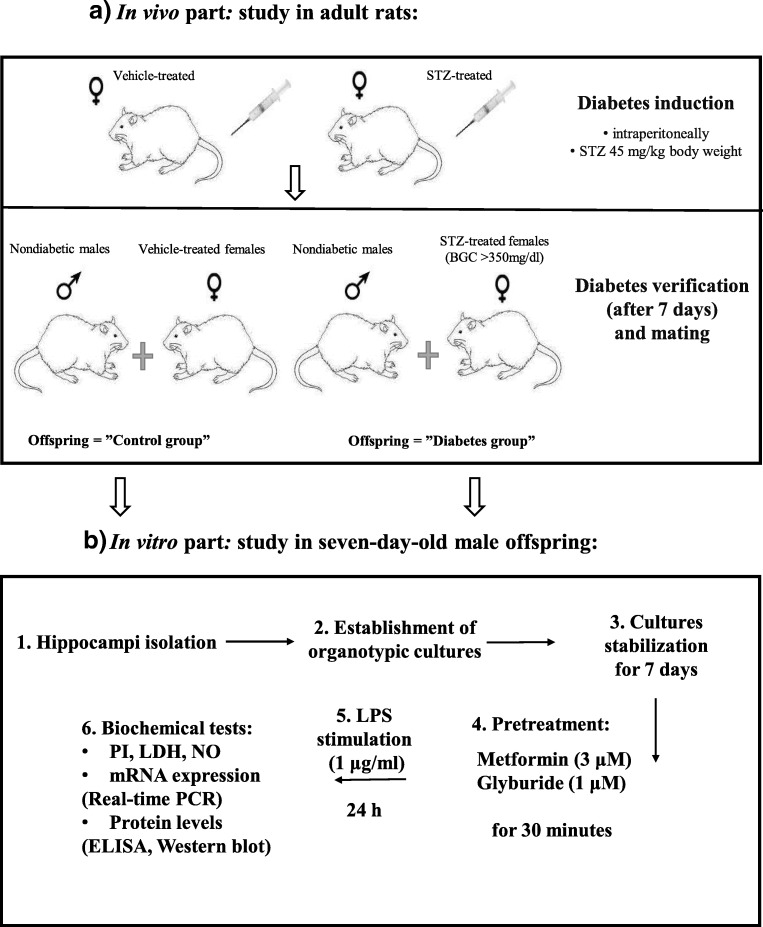
Schematic showing the experimental design

A schematic showing the experimental design is illustrated in Fig. 1.

### Fluorescence-Activated Cell Sorting Study

Twenty-four hours after LPS stimulation, the hippocampal organotypic cultures (groups: control, diabetes, control + LPS, diabetes + LPS) were stained with propidium iodide (PI). PI does not cross the cell membrane but instead stains DNA released from the cells of which the membranes disintegrate. Briefly, hippocampal cultures were transferred to 1.5-ml centrifuge tubes containing 500 μl of ice-cold HBSS. After 5 min of centrifugation (800 rpm), the cultures were incubated with 500 μl of prewarmed collagenase A solution (4 mg/ml; 30 min at 37 °C; Biomed, Poland), centrifuged, and incubated again with 500 μl of prewarmed trypsin (0.05%; 20 min at 37 °C; ThermoFisher Scientific, USA). Next, the cultures were stained with a PI solution (100 ng/ml in PBS; Sigma Aldrich, Germany) for 5 min at room temperature. The hippocampal cells were analyzed using the BD Fluorescence-Activated Cell Sorting (FACS) Canto II System and BD FACS Diva™ v5.0.1 Software (BD Biosciences, USA) in the fluorescence channel for PE (phycoerythrin, red fluorescence). The PI-negative cells were considered to be undamaged and alive, while the PI-positive cells were considered to be dead.

### Determination of Lactate Dehydrogenase Activity

Twenty-four hours after culture treatment, lactate dehydrogenase (LDH) activity was measured in the culture medium using a colorimetric method (Cytotoxicity Detection Kit, Roche Diagnostic, Germany). The intensity of red fluorescence formed in the assay, measured at a wavelength of 490 nm using the Infinite 200 PRO Detector system (TECAN, Switzerland), was proportional to LDH activity and to the number of damaged cells.

### Nitric Oxide Release Assay

Nitric oxide (NO) secreted in the culture medium was measured by the Griess reaction. After 24 h of treatment of the hippocampal cultures, 50 μl of medium from each well was collected and mixed with an equal volume of Griess reagent (0.1% N-1-naphthylethylenediamine dihydrochloride and 1% sulfanilamide in 5% phosphoric acid; Sigma Aldrich, Germany) in a 96-well plate. The absorbance was measured immediately at 540 nm using the Infinite 200 PRO Detector system (TECAN, Switzerland).

### Gene Expression Study

#### RNA Extraction and cDNA Preparation

Freshly isolated hippocampal tissue samples (each sample was collected from two wells) were immediately placed in an RNAlater® solution (Applied Biosystems, USA) and stored at − 20 °C prior to total RNA extraction. Total RNA was extracted using an RNeasy Mini Kit (Qiagen, Germany) following the manufacturer’s instructions. The RNA concentration was measured using a NanoDrop ND-1000 Spectrometer (Thermo Scientific, Wilmington, USA). The RNA was reverse transcribed into cDNA using a commercial kit for reverse transcription (Applied Biosystems, USA) according to the manufacturer’s instructions.

#### Real-Time PCR

Real-time PCR was performed using TaqMan probes and primers for the genes *il-1β* (Rn00580432_m1), *il-18* (Rn01422083_m1), *il-6* (Rn01410330_m1), *tnf-α* (Rn00562055_m1), *nlrp3* (Rn04244625), *asc* (Rn00597229_g1), and *casp1* (Rn00562724_m1) (all obtained from ThermoFisher Scientific, USA) and the FastStart Universal Probe Master (Rox) kit (Roche Diagnostic, Germany). Amplification was performed using a 20-μl mixture containing PCR master mix, the cDNA used as the PCR template, TaqMan forward and reverse primers, and 250 nM of a hydrolysis probe labeled at the 5′ end with the fluorescent reporter FAM and at the 3′ end with a quenching dye. The thermal cycling conditions were 2 min at 50 °C and 10 min at 95 °C, followed by 40 cycles at 95 °C for 15 s and 60 °C for 1 min. The samples were run in a CFX96 Real-Time System (BIO-RAD, Hercules, CA, USA). The threshold value (Ct) for each sample was set in the exponential phase of PCR, and the ΔΔCt method was used for data analysis. *Rplp0* (Rn03302271_gH) (ThermoFisher Scientific, USA) was used as the reference gene.

### Enzyme-Linked Immunosorbent Assays

The concentrations of IL-1β, IL-18, IL-6, TNF-α, TLR-4, NF-κB p65, NF-κB p65 (phospho), NLRP3, and caspase-1 in the slice homogenates and of IL-1β, IL-18, IL-6, TNF-α in the medium were determined by enzyme-linked immunosorbent assays (ELISAs) using commercially available kits (IL-1β and IL-18 kits were obtained from ThermoFisher Scientific, USA; IL-6 and TNF-α kits were obtained from Becton Dickinson, USA; TLR-4 and NF-κB p65 kits were obtained from Cusabio China; an NF-κB p65 (phospho) kit was obtained from ThermoFisher Scientific; an NLRP3 kit was obtained from Cloud-Clone Corp., USA; and a caspase-1 kit was obtained from EiaB Science, China) according to the manufacturers’ instructions. Briefly, standards or probes (50 or 100 μl) were dispensed into 96 wells coated with rat IL-1β, IL-18, IL-6, TNF-α, TLR-4, NF-kB p65, NF-kB p65 (phospho), NLRP3, or caspase-1 antibodies and incubated. After extensive washing, HRP-conjugated streptavidin was pipetted into the wells and incubated. The wells were washed, and 3,3′,5,5′-tetramethylbenzidine (TMB) was added. The color developed in proportion to the concentration of the measured protein. Each reaction was stopped after the addition of a stop solution. The absorbance was measured using the Infinite 200 PRO Detector system (TECAN, Switzerland) set to the appropriate wavelength. The detection limits were as follows: IL-1β < 39 pg/ml, IL-18 < 4 pg/ml, IL-6 < 78 pg/ml, TNF-α < 31.3 pg/ml, TLR-4 < 0.156 ng/ml, NF-κB p65 < 1.56 ng/ml, NF-κB p65 (phospho) not applicable, NLRP3 < 0.112 ng/ml, and caspase-1 < 7.88 pg/ml. The intra- and interassay precision was dependent on the properties of the assay.

### Western Blot Analysis

Western blot analysis was conducted to determine the level of the ASC protein. The cultures were lysed in RIPA buffer (Sigma-Aldrich, USA) containing protease inhibitors (ThermoFisher Scientific, USA). Samples containing equal amounts of total protein were mixed with gel loading buffer (Bio-Rad, Hercules, CA, USA) in a 4:1 ratio (*v*/*v*) and boiled for 6 min at 96 °C. The proteins were separated by SDS–PAGE (4–20% gel; Bio-Rad, Hercules, CA, USA) under constant voltage (200 V) and transferred to PVDF membranes (Trans-Blot Turbo; Bio-Rad, Hercules, CA, USA). After transfer, the membranes were cut to allow simultaneous incubation with two antibodies (ASC and β-actin). Then, the membranes were blocked using 5% bovine serum albumin (Sigma Aldrich, USA) for 1 h and incubated with an anti-ASC primary antibody (Santa Cruz Biotechnology, Inc., Dallas, TX, USA) or an anti-β-actin primary antibody (Sigma Aldrich, USA) overnight at 4 °C. The blots were washed four times with TBS containing 0.1% Twsseen-20 (TBST) and then incubated with appropriate secondary antibodies (Vector Laboratories, UK) for 1 h at room temperature. After washing (4 × 10 min in TBST), the bands were developed using BM Chemiluminescence Western Blotting Substrate (POD) (Roche, Germany). The protein band intensities were normalized to β-actin as an internal loading control. The relative levels of immunoreactivity were densitometrically quantified using Fujifilm Multi Gauge software (Fujifilm, Japan).

### Statistical Analysis

All biochemical experiments were carried out under exactly the same conditions for every sample, regardless of the type of treatment.

The results were analyzed using the STATISTICA 10 program. The subsequent statistical analyses used factorial analyses of variance (ANOVA) to determine the effects of the factors followed, when appropriate, by Duncan’s *post hoc* test. A *p* value < 0.05 was considered to indicate significance.

All graphs were prepared using GraphPad Prism 5.

## Results

### The Impact of Maternal Diabetes on Propidium Iodide Uptake in Basal and LPS-Stimulated Hippocampal Organotypic Cultures (Assayed by the Fluorescence-Activated Cell Sorting Method in Slices)

**Fig. 2:**
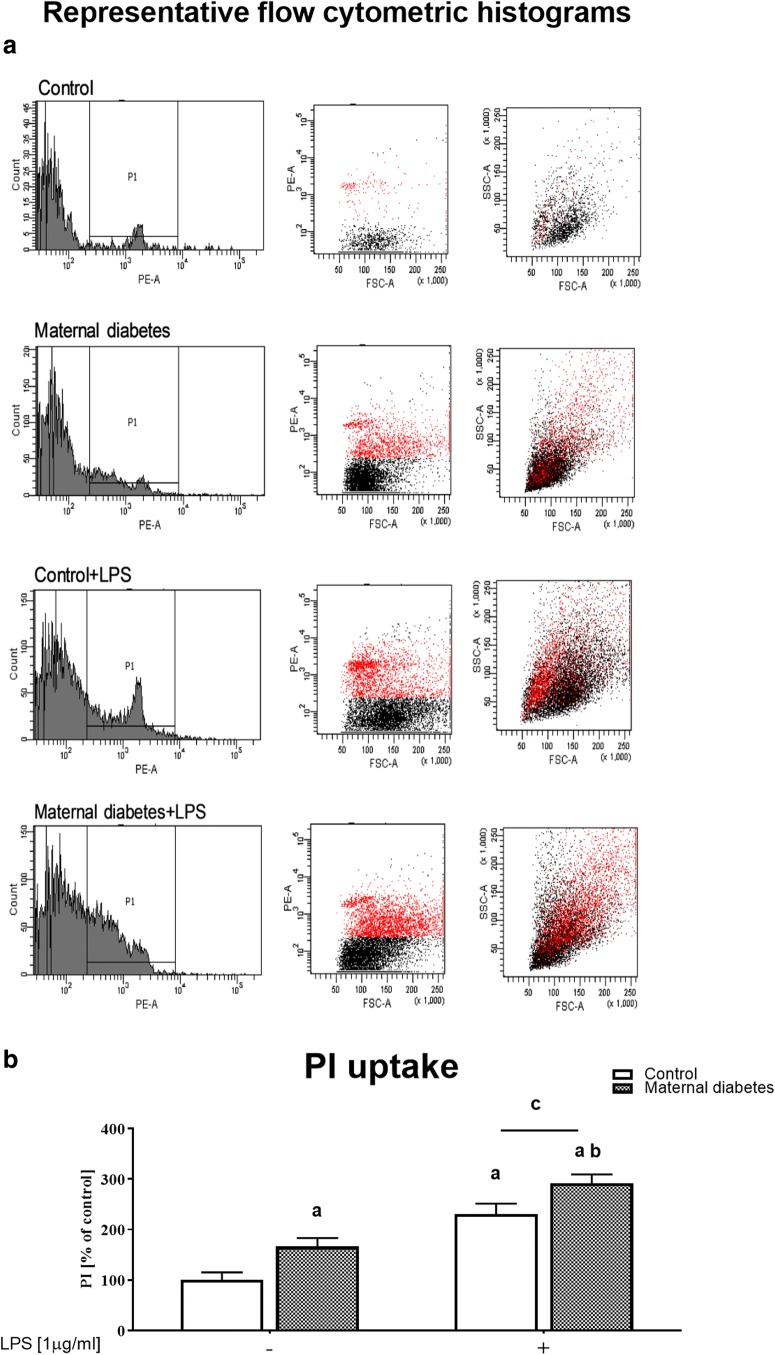
The impact of maternal diabetes on PI uptake in basal and LPS-stimulated hippocampal organotypic cultures (OHCs) (assayed by the FACS method). **a** Representative flow cytometric histograms. **b** Illustration of the percentage of PI-positive cells in the examined groups. Control and diabetes OHCs were stimulated for 24 h with lipopolysaccharide (LPS; 1 μg/ml). The results are expressed as the mean percentages relative to the control ± SEM. The data are from two independent experiments: **a***p* < 0.05 vs. control. **b***p* < 0.05 vs. diabetes. **c***p* < 0.05 vs. control + LPS. PI—propidium iodide

In the first stage of the experiments, hippocampal organotypic cultures (control and diabetic) were stained with propidium iodide (PI). Maternal diabetes increased cell death processes (*p* < 0.05), as presented in Fig. 2. In addition, a post hoc analysis showed that diabetic cultures stimulated with LPS (1 μg/ml, 24 h) were characterized by intensified PI staining (*p* < 0.05) compared with controls.

### The Impact of Lipopolysaccharide and/or Antidiabetic Drugs on Cell Death in Hippocampal Organotypic Cultures Obtained from the Offspring of Control and Diabetic Dams (Measured by the Lactate Dehydrogenase Test in the Culture Medium)

**Fig. 3:**
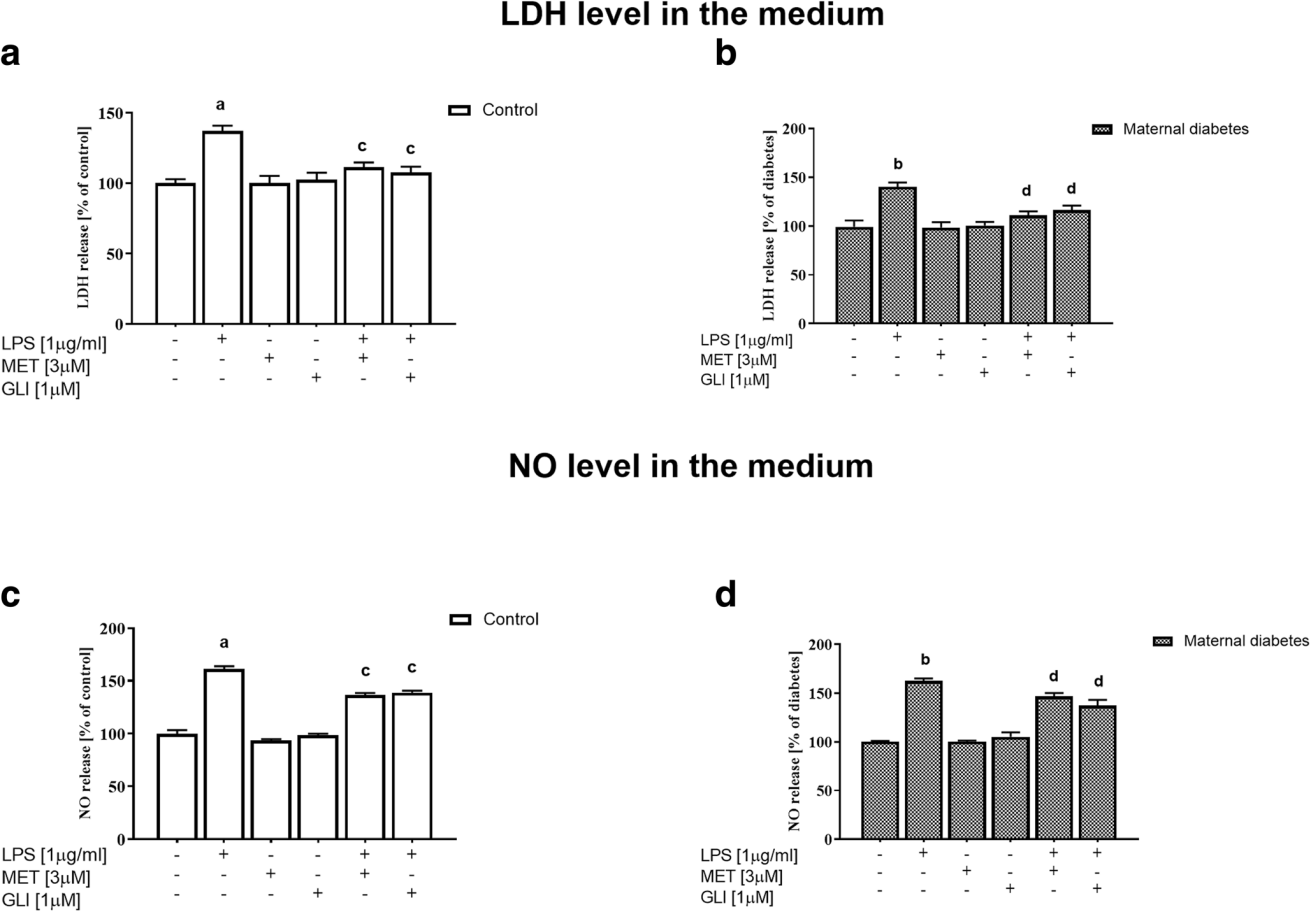
The impact of LPS and/or antidiabetic drugs on **a**, **b** LDH and **c**, **d** NO release in hippocampal organotypic cultures obtained from the offspring of control and diabetic dams (OHCs). OHCs were pretreated for 30 min with MET (3 μM) or GLI (1 μM) and then stimulated with lipopolysaccharide (LPS; 1 μg/ml) for 24 h. Control cultures were treated with the appropriate vehicle. The results are expressed as the mean percentages relative to the control (**a**, **c**) or diabetes (**b**, **d**) ± SEM. The data are from three independent experiments. **a***p* < 0.05 vs. control. **b***p* < 0.05 vs. diabetes. **c***p* < 0.05 vs. control + LPS. **d***p* < 0.05 vs. diabetes + LPS. LDH—lactate dehydrogenase; NO—nitric oxide

In the next set of experiments, we examined the effect of LPS in hippocampal organotypic cultures to confirm the results obtained in the flow cytometry study and to check whether the applied antidiabetic drugs (metformin/glyburide) have protective properties against LPS-induced changes. We confirmed the harmful effect of LPS (1 μg/ml, 24 h) observed in the flow cytometry study; in both the control and diabetes cultures, increased LDH release was demonstrated (*p* < 0.05) (Fig. 3a, b). Experiments performed on control and diabetic cultures showed that, under basal conditions (without LPS stimulation), neither metformin (3 μM, 24 h) nor glyburide (1 μM, 24 h) had any effect on LDH release, as shown in Fig. 3a and b. Glyburide (1 μM, 24 h) and metformin (3 μM, 24 h) diminished LPS-evoked LDH release (*p* < 0.05).

### The Impact of Lipopolysaccharide and/or Antidiabetic Drugs on Nitric Oxide Release in Hippocampal Organotypic Cultures Obtained from the Offspring of Control and Diabetic Dams (Measured by the Griess Method in the Culture Medium)

We found that stimulation with LPS (1 μg/ml, 24 h) significantly increased NO levels in the medium of the control (*p* < 0.05) and diabetic (*p* < 0.05) cultures, as shown in Fig. 3c and d. Experiments performed on control and diabetic cultures showed that, under basal conditions, neither metformin (3 μM, 24 h) nor glyburide (1 μM, 24 h) had any effect on NO release, as shown in Fig. 3c and d. Glyburide (1 μM, 24 h) and metformin (3 μM, 24 h) pretreatment decreased (*p* < 0.05) NO release from hippocampal control and diabetic cultures stimulated with LPS (1 μg/ml, 24 h).

#### The Impact of Maternal Diabetes and/or Lipopolysaccharide Stimulation on the Gene Expression of Pro-Inflammatory Factors (*il-1β*, *il-18*, *il-6*, *tnf-α*) and on NLRP3 Inflammasome Subunits (*nlrp3*, *asc*, *casp1*) in the Homogenates of Hippocampal Organotypic Cultures (Measured by Real-Time PCR)

Maternal diabetes did not have any effect on the gene expression of the investigated factors, except for that of *il-18* (Table 1)*.* With regard to this cytokine, we demonstrated that maternal diabetes diminished the expression of *il-18* in the offspring hippocampi (*p* < 0.05).

**Table 1:** The impact of maternal diabetes and/or LPS stimulation on the gene expression of the pro-inflammatory factors: *il-1β*, *il-18*, *il-6*, *tnf-α*, and the NLRP3 inflammasome subunits: *nlrp3*, *asc*, and *casp1* in hippocampal organotypic cultures (OHCs) (measured by real-time PCR). OHCs were stimulated with lipopolysaccharide (LPS; 1 μg/ml) for 24 h. Control cultures were treated with the appropriate vehicle. The results are expressed as the average fold change ± SEM. The data are from three independent experiments. *asc* apoptosis-associated speck-like protein containing a caspase recruitment domain, *casp1* caspase-1, *il* interleukin, *nlrp3* Nod-like receptor pyrin-containing 3 subunit, *tnf-α* tumor necrosis factor α

Gene expression				
	Control	Control LPS	Diabetes	Diabetes LPS
*il-1β*	1.04 ± 0.14	107.44 ± 19.70^a^	0.72 ± 0.13	165.40 ± 50.36^ab^
*il-18*	1.02 ± 0.10	1.76 ± 0.22^a^	0.43 ± 0.09^a^	0.71 ± 0.10^c^
*il-6*	1.04 ± 0.15	470.89 ± 128.57^a^	0.96 ± 0.11	380.49 ± 80.04^ab^
*tnf-α*	1.08 ± 0.18	6.72 ± 1.37^a^	0.61 ± 0.11	3.11 ± 0.30^bc^
*nlrp3*	1.00 ± 0.04	1.95 ± 0.21^a^	0.85 ± 0.11	1.58 ± 0.18^ab^
*asc*	1.02 ± 0.08	0.93 ± 0.11	0.82 ± 0.07	0.75 ± 0.07^a^
*casp1*	1.01 ± 0.05	1.95 ± 0.21^a^	0.75 ± 0.07	1.67 ± 0.18^ab^

^a^*p* < 0.05 vs. control

^b^*p* < 0.05 vs. diabetes

^c^*p* < 0.05 vs. control + LPS

A stimulatory effect of LPS (1 μg/ml, 24 h) on the examined genes, except for *asc*, was observed in the control group (*p* < 0.05). In the diabetic group, LPS stimulation (1 μg/ml, 24 h) increased *il-1β*, *il-6*, *tnf-α*, *nlrp3*, and *casp1* mRNA expression but did not change the expression of *il-18* or *asc.* Additionally, we found that, in the case of *tnf-α*, the effect of LPS was significantly lower in the diabetic cultures than in the control.

#### The Impact of Antidiabetic Drugs on the Gene Expression of Pro-Inflammatory Factors (*il-1β*, *il-18*, *il-6*, *tnf-α*) and NLRP3 Inflammasome Subunits (*nlrp3*, *asc*, *casp1*) in the Homogenates of Hippocampal Organotypic Cultures (Measured by Real-Time PCR)

In control OHCs, neither metformin nor glyburide had any effect on the gene expression of the investigated factors (Table 2). In diabetes, we observed an increase in *il-1β* after metformin treatment (3 μM, 24 h) (*p* < 0.05). Moreover, we demonstrated a decrease in *tnf-α* and *il-18* after glyburide treatment (1 μM, 24 h) (vs. control + GLI) and a decrease in *il-18*, while *il-1β* increased after metformin treatment in the diabetic rats vs. the metformin-treated control group.

**Table 2:** The impact of antidiabetic drugs on the gene expression of the pro-inflammatory factors: *il-1β*, *il-18*, *il-6*, *tnf-α*, and the NLRP3 inflammasome subunits: *nlrp3*, *asc*, and *casp1* in control and diabetic hippocampal organotypic cultures (OHCs) (measured by real-time PCR). OHCs were treated for 30 min with MET (3 μM) or GLI (1 μM). Control cultures were treated with the appropriate vehicle. The results are expressed as the average fold change ± SEM. The data are from three independent experiments. *asc* apoptosis-associated speck-like protein containing a caspase recruitment domain, *casp1* caspase-1, *il* interleukin, *nlrp3* Nod-like receptor pyrin-containing 3 subunit, *tnf-α* tumor necrosis factor α

Gene expression						
	Control	Control Metformin	Control Glyburide	Diabetes	Diabetes Metformin	Diabetes Glyburide
*il-1β*	1.04 ± 0.14	0.92 ± 0.12	0.99 ± 0.31	0.72 ± 0.13	26.40 ± 12.12^abc^	1.14 ± 0.29
*il-18*	1.02 ± 0.10	0.78 ± 0.12	0.89 ± 0.13	0.43 ± 0.09 ^a^	0.44 ± 0.08^ac^	0.40 ± 0.03^ad^
*il-6*	1.04 ± 0.15	0.72 ± 0.11	1.07 ± 0.16	0.96 ± 0.11	0.67 ± 0.11	1.12 ± 0.25
*tnf-α*	1.08 ± 0.18	1.19 ± 0.27	1.29 ± 0.08	0.61 ± 0.11	0.69 ± 0.18	0.64 ± 0.04^d^
*nlrp3*	1.00 ± 0.04	0.88 ± 0.08	1.02 ± 0.17	0.85 ± 0.11	0.90 ± 0.15	0.78 ± 0.12
*asc*	1.02 ± 0.08	0.89 ± 0.04	1.06 ± 0.13	0.82 ± 0.07	0.85 ± 0.10	0.85 ± 0.14
*casp1*	1.01 ± 0.05	0.82 ± 0.04	1.08 ± 0.28	0.75 ± 0.07	0.84 ± 0.11	0.77 ± 0.12

^a^*p* < 0.05 vs. control

^b^*p* < 0.05 vs. diabetes

^c^*p* < 0.05 vs. control + MET

^d^*p* < 0.05 vs. control + GLI

#### The Impact of Antidiabetic Drugs on the Gene Expression of Pro-inflammatory Factors (*Il-1β, Il-18, Il-6, tnf-α*) and NLRP3 Inflammasome Subunits (*nlrp3*, *asc*, *casp1*) in the Homogenates of Control and LPS-Stimulated Diabetic Hippocampal Organotypic Cultures (Measured by Real-Time PCR)

Both metformin (3 μM, 24 h) and glyburide (1 μM, 24 h) did not normalize the LPS-induced changes in the gene expression of *il-1β*, *il-18*, *il-6*, *nlrp3*, and *casp1* in the control and diabetic groups (Table 3). However, *tnf-α* gene expression was significantly lower in the metformin/LPS/diabetic group vs. the metformin/LPS/control group, and *il-18* was significantly reduced in the glyburide/LPS/diabetic group compared to the glyburide/LPS/control group.

**Table 3:** The impact of antidiabetic drugs on the gene expression of the pro-inflammatory factors: *il-1β*, *il-18*, *il-6*, and *tnf-α* and the NLRP3 inflammasome subunits: *nlrp3*, *asc*, and *casp1* in LPS-stimulated control and diabetic hippocampal organotypic cultures (OHCs) (measured by real-time PCR). OHCs were pretreated for 30 min with MET (3 μM) or GLI (1 μM) and then stimulated with lipopolysaccharide (LPS; 1 μg /ml) for 24 h. The results are expressed as the average fold change ± SEM. The data are from three independent experiments. *Asc* apoptosis-associated speck-like protein containing a caspase recruitment domain, c*asp1* caspase-1, *il* interleukin, *nlrp3* Nod-like receptor pyrin-containing 3 inflammasome, *tnf-α* tumor necrosis factor α

Gene expression						
	Control LPS	Control LPS metformin	Control LPS Glyburide	Diabetes LPS	Diabetes LPS Metformin	Diabetes LPS Glyburide
*il-1β*	107.44 ± 19.70	78.25 ± 8.60	94.05 ± 6.22	165.40 ± 50.36	183.09 ± 85.68	109.47 ± 35.33
*il-18*	1.76 ± 0.22	1.57 ± 0.22	2.33 ± 0.25	0.71 ± 0.10^a^	1.06 ± 0.17	0.90 ± 0.17^b^
*il-6*	470.89 ± 128.57	546.20 ± 138.65	625.17 ± 217.91	380.49 ± 80.04	331.00 ± 49.75	347.87 ± 73.49
*tnf-α*	6.72 ± 1.37	6.50 ± 1.85	6.39 ± 1.78	3.11 ± 0.30^a^	2.28 ± 0.45^c^	4.35 ± 0.62
*nlrp3*	1.95 ± 0.21	1.95 ± 0.26	1.99 ± 0.40	1.58 ± 0.18	1.78 ± 0.34	1.71 ± 0.32
*asc*	0.93 ± 0.11	0.75 ± 0.11	0.97 ± 0.12	0.75 ± 0.07	0.86 ± 0.11	0.74 ± 0.04
*casp1*	1.95 ± 0.21	1.74 ± 0.28	1.86 ± 0.29	1.67 ± 0.18	2.04 ± 0.27	1.67 ± 0.11

^a^*p* < 0.05 vs. control + LPS

^b^*p* < 0.05 vs. control + LPS + glyburide

^c^*p* < 0.05 vs. control + LPS + metformin

### The Impact of Maternal Diabetes and/or Antidiabetic Drugs on Pro-Inflammatory Factor Levels in Basal and LPS-Stimulated Hippocampal Organotypic Cultures (Assayed by ELISA)

1. **IL-1β**

#### Protein Level in the Homogenates of Hippocampal Organotypic Cultures

Neither maternal diabetes nor LPS (1 μg/ml, 24 h) had any effect on the protein level of IL-1β in the homogenates (Table 4). In hippocampal homogenates from control cultures stimulated with LPS (1 μg/ml, 24 h) and pretreated with either metformin (3 μM, 24 h) or glyburide (1 μM, 24 h), diminished levels of cytokines (*p* < 0.05) in comparison to those in the control cultures treated with either drug were observed. Metformin treatment (3 μM; 24 h) in the control cultures increased the level of IL-1β (*p* < 0.05), but this effect was not observed in the diabetic cultures.

**Table 4:** The impact of maternal diabetes and/or antidiabetic drugs (metformin (MET) or glyburide (GLI)) on IL-1β, IL-18, IL-6, and TNF-α protein levels in homogenates of basal and LPS-stimulated hippocampal organotypic cultures (OHCs). OHCs were pretreated for 30 min with MET (3 μM) or GLI (1 μM) and then stimulated with lipopolysaccharide (LPS; 1 μg /ml) for 24 h. Control cultures were treated with the appropriate vehicle. The results are expressed as the means ± SEM. The data are from three independent experiments. *IL* interleukin, *TNF-α* tumor necrosis factor α

Cytokine protein levels in homogenates of OHCs												
	Control	Diabetes	Control LPS	Diabetes LPS	Control metformin	Diabetes metformin	Control glyburide	Diabetes glyburide	Control LPS metformin	Diabetes LPS metformin	Control LPS glyburide	Diabetes LPS glyburide
IL-1β [pg/mg protein]	1119.6 ± 97.5	580.8 ± 238.7	1196.2 ± 107.3	777.2 ± 110.9	3243.0 ± 612.3 ^a^	726.1 ± 27.6	1730.4 ± 393.7	860.1 ± 121.5	1287.9 ± 170.0 ^d^	558.5 ± 69.9	557.4 ± 103.6 ^e^	230.3 ± 30.9
IL-18 [pg/mg protein]	253.8 ± 19.6	369.2 ± 32.7 ^a^	222.0 ± 19.4	184.7 ± 24.8 ^b^	397.4 ± 15.2 ^a^	332.5 ± 43.5	359.2 ± 28.2 ^a^	352.6 ± 27.0	235.0 ± 8.1	241.5 ± 3.4	330.6 ± 70.0 ^c^	256.2 ± 11.3
IL-6 [pg/mg protein]	6699.0 ± 58.8	10,341.3 ± 891.0 ^a^	9832.4 ± 1202.5 ^a^	9616.4 ± 1108.7	9294.7 ± 601.9	9516.9 ± 731.8	7867.4 ± 576.7	10,250.3 ± 306.4	9183.0 ± 1186.5	8847.9 ± 1032.8	9711.0 ± 362.4	8239.3 ± 960.3
TNF-α [pg/mg protein]	17.9 ± 3.5	21.3 ± 4.2	44.6 ± 2.1^a^	33.4 ± 7.3	16.7 ± 0.2	25.2 ± 3.0	22.6 ± 5.8	22.8 ± 8.5	43.7 ± 9.0 ^a^	39.4 ± 9.7	51.8 ± 4.9 ^a^	31.8 ± 11.0

^a^*p* < 0.05 vs. control

^b^*p* < 0.05 vs. diabetes

^c^*p* < 0.05 vs. control + LPS

^d^*p* < 0.05 vs. control + MET

^e^*p* < 0.05 vs. control + GLI

#### Protein Level in the Medium of Hippocampal Organotypic Cultures

**Fig. 4:**
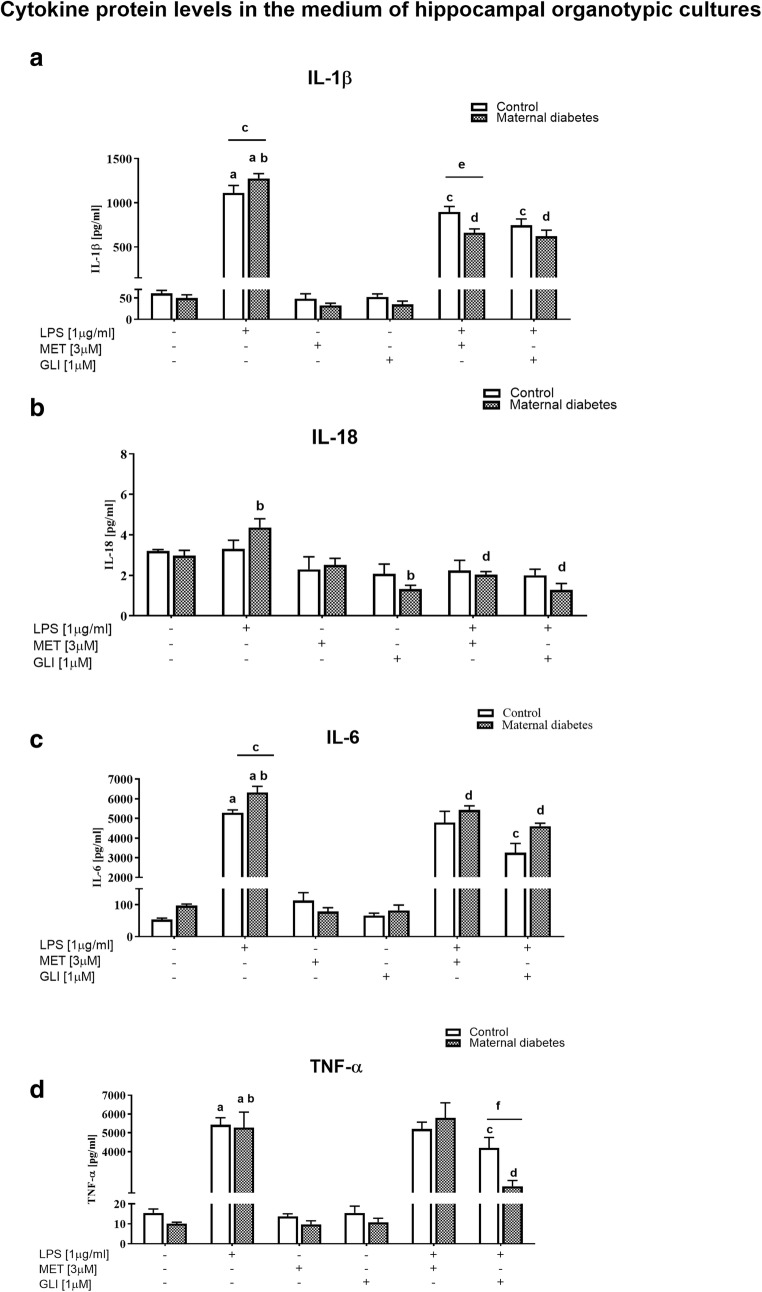
The impact of maternal diabetes and/or antidiabetic drugs (metformin (MET) or glyburide (GLI)) on **a** IL-1β, **b** IL-18, **c** IL-6, and **d** TNF-α levels in the medium of basal and LPS-stimulated hippocampal organotypic cultures (OHCs) (assayed by ELISA). OHCs were pretreated for 30 min with MET (3 μM) or GLI (1 μM) and then stimulated with lipopolysaccharide (LPS; 1 μg/ml) for 24 h. Control cultures were treated with the appropriate vehicle. The results are expressed as the means ± SEM. The data are from three independent experiments. **a***p* < 0.05 vs. control. **b***p* < 0.05 vs. diabetes; **c***p* < 0.05 vs. control + LPS. **d***p* < 0.05 vs. diabetes + LPS. **e***p* < 0.05 vs. control + LPS + MET. **f***p* < 0.05 vs. control + LPS + GLI. IL—interleukin; TNF-α—tumor necrosis factor α

LPS treatment (1 μg/ml, 24 h) significantly increased the secretion of IL-1β in the control (*p* < 0.05) and diabetic cultures (*p* < 0.05) (Fig. 4a). Furthermore, the LPS-stimulated diabetic cultures secreted more IL-1β than the control cultures (*p* < 0.05). Although no effect of metformin or glyburide was found on this parameter under basal conditions, we demonstrated that both metformin (3 μM, 24 h) and glyburide (1 μM, 24 h) pretreatment significantly (*p* < 0.05) inhibited the secretion of IL-1β into the medium from control and diabetic cultures stimulated with LPS. The inhibitory effect of metformin (3 μM, 24 h) was stronger in diabetic hippocampi than in controls (*p* < 0.05).

2. **IL-18**

#### Protein Level in the Homogenates of Hippocampal Organotypic Cultures

In diabetic hippocampi, the IL-18 concentration was higher than that in the control (*p* < 0.05). LPS stimulation (1 μg/ml, 24 h) diminished the level of IL-18 only in diabetic hippocampi (*p* < 0.05) (Table 4). Glyburide (1 μM, 24 h) pretreatment increased (*p* < 0.05) the concentration of IL-18 in the control cultures stimulated with LPS (1 μg/ml, 24 h), while this effect was not observed in the diabetic cultures. Under basal conditions, metformin (3 μM, 24 h) and glyburide (3 μM, 24 h) caused an increase in the level of this cytokine in the control cultures (*p* < 0.05).

#### Protein Level in the Medium of Hippocampal Organotypic Cultures

LPS (1 μg/ml, 24 h) upregulated (*p* < 0.05) the level of IL-18 in the medium of diabetic hippocampal cultures, and both drugs normalized this effect (*p* < 0.05). Moreover, under basal conditions in diabetic cultures, glyburide (1 μM, 24 h) was able to reduce the secretion of IL-18 (p < 0.05) (Fig. 4b ).

3. **IL-6**

#### Protein Level in the Homogenates of Hippocampal Organotypic Cultures

Diabetes significantly enhanced the level of IL-6 in comparison to that in the control (*p* < 0.05), while LPS treatment (1 μg/ml, 24 h) increased IL-6 only in the control cultures (Table 4).

#### Protein Level in the Medium of Hippocampal Organotypic Cultures

LPS stimulation (1 μg/ml, 24 h) significantly increased (*p* < 0.05) the level of IL-6 in the medium of both the control and diabetic cultures (Fig. 4c). Moreover, this effect was higher in the diabetic cultures (*p* < 0.05). Metformin (3 μM, 24 h) and glyburide (1 μM, 24 h) pretreatment weakened the effect of LPS (1 μg/ml, 24 h) in diabetes (*p* < 0.05), while in the control group, this effect was observed only with regard to glyburide (*p* < 0.05). There was no effect of the drugs under basal conditions in either of the examined groups (control and diabetes).

4. **TNF-α**

#### Protein Level in the Homogenates of Hippocampal Organotypic Cultures

Only in the cultures derived from control offspring and stimulated with LPS (1 μg/ml, 24 h) was a statistically significant increase (*p* < 0.05) in TNF-α concentration observed (Table 4). Pretreatment with metformin (3 μM, 24 h) or glyburide (1 μM, 24 h) did not prevent the LPS-induced upregulation of this factor in the control cultures.

#### Protein Level in the Medium of Hippocampal Organotypic Cultures

Stimulation with LPS (1 μg/ml, 24 h) led to increased TNF-α levels in the medium of both control and diabetic hippocampal cultures (Fig. 4d). Metformin (3 μM, 24 h) and glyburide (1 μM, 24 h) treatment did not affect TNF-α levels under basal conditions. Glyburide (1 μM; 24 h) pretreatment significantly diminished the level of TNF-α in the hippocampi stimulated with LPS in both the control (*p* < 0.05) and diabetic (*p* < 0.05) cultures. Importantly, the effect observed in the diabetic cultures was significantly stronger than that observed in the control cultures (*p* < 0.05).

### The Impact of Maternal Diabetes and/or Antidiabetic Drugs on TLR-4 and Phospho-p65/Total p65 Protein Levels in Basal and LPS-Stimulated Hippocampal Organotypic Cultures (Assayed by ELISA)

1. **TLR-4**

#### Protein Level in the Homogenates of Hippocampal Organotypic Cultures

**Fig. 5:**
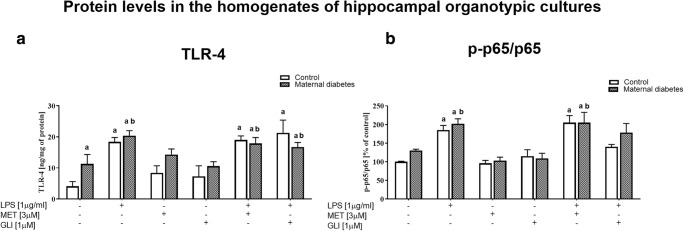
The impact of maternal diabetes and/or antidiabetic drugs (metformin (MET) or glyburide (GLI)) on **a** TLR-4 and **b** phospho-p65/total p65 protein levels in basal and LPS-stimulated hippocampal organotypic cultures (OHCs) (assayed by ELISA). OHCs were pretreated for 30 min with MET (3 μM) or GLI (1 μM) and then stimulated with lipopolysaccharide (LPS; 1 μg/ml) for 24 h. Control cultures were treated with the appropriate vehicle. The results are expressed as **a** the means ± SEM and **b** the mean percentages relative to the control ± SEM. The data are from three independent experiments. **a***p* < 0.05 vs. control; **b***p* < 0.05 vs. diabetes. TLR-4—Toll-like receptor 4

In the diabetic cultures, there was an increase in the level of TLR-4 in comparison to that in the control (*p* < 0.05) (Fig. 5a). There was no effect of metformin (3 μM, 24 h) or glyburide (1 μM, 24 h) in the control and diabetic cultures (under basal conditions). LPS (1 μg/ml, 24 h) significantly increased the TLR-4 protein level in both the control and diabetic groups (*p* < 0.05). The drugs used had no effect on the observed LPS-induced upregulation of TLR-4.

2. **Phospho-p65/total p65 protein level ratio**

#### Protein Levels in the Homogenates of Hippocampal Organotypic Cultures

An increase in the phospho-p65/total p65 ratio was observed after LPS stimulation (1 μg/ml, 24 h) in the control and diabetic cultures (*p* < 0.05) (Fig. 5b). Metformin (3 μM, 24 h) did not normalize the LPS-evoked increase in the phospho-p65/total p65 ratio. Pretreatment with glyburide (1 μM, 24 h) prevented (*p* < 0.05) the LPS-induced upregulation of this factor (in both the control and diabetic cultures).

### The Impact of Maternal Diabetes and/or Antidiabetic Drugs on NLRP3, ASC, and Caspase-1 Protein Levels in Basal and LPS-Stimulated Hippocampal Organotypic Cultures (Measured by ELISA and Western Blot)

1. **NLRP3**

#### Protein Level in the Homogenates of Hippocampal Organotypic Cultures

**Fig. 6:**
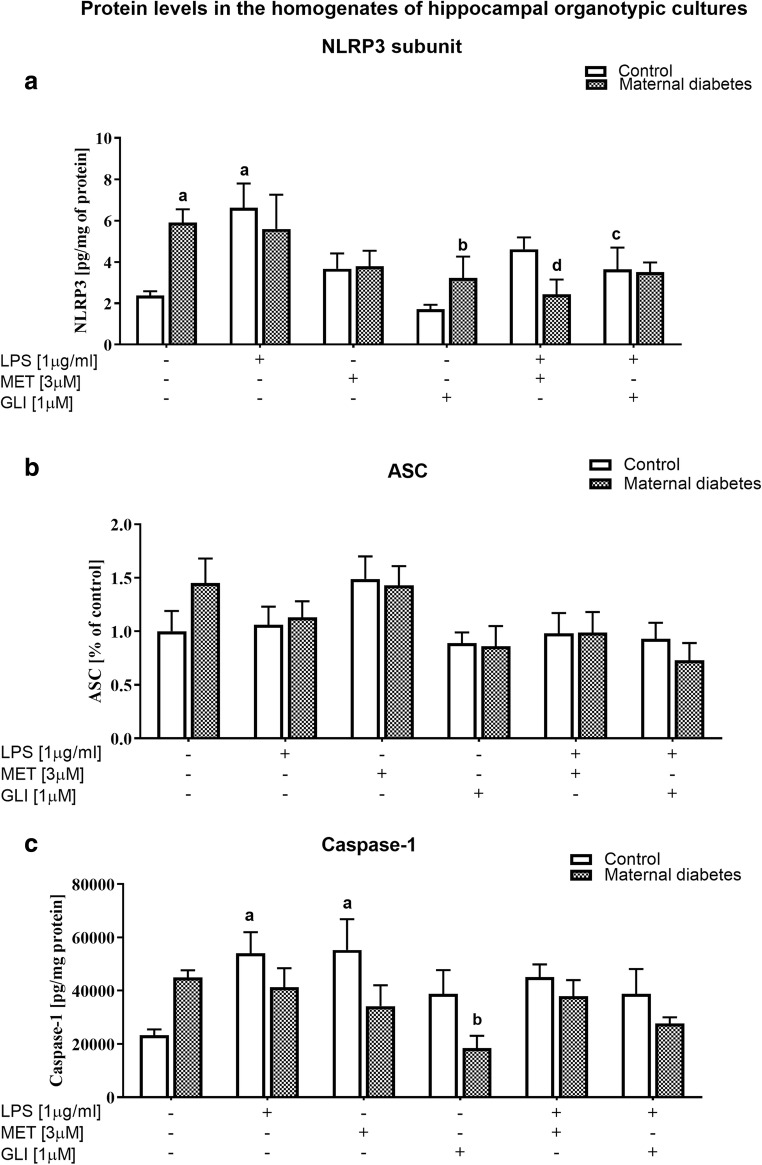
The impact of maternal diabetes and/or antidiabetic drugs (metformin (MET) or glyburide (GLI)) on **a** NLRP3, **b** ASC, and **c** caspase-1 protein levels in basal and LPS-stimulated hippocampal organotypic cultures (OHCs) (assayed by ELISA/WB). OHCs were pretreated for 30 min with MET (3 μM) or GLI (1 μM) and then stimulated with lipopolysaccharide (LPS; 1 μg /ml) for 24 h. Control cultures were treated with the appropriate vehicle. The results are expressed as **a**, **c** the means ± SEM and **b** the mean percentages relative to the control ± SEM. The data are from three independent experiments. **a***p* < 0.05 vs. control. **b***p* < 0.05 vs. diabetes. **c***p* < 0.05 vs. control + LPS. **d***p* < 0.05 vs. diabetes + LPS. ASC—apoptosis-associated speck-like protein containing a caspase recruitment domain; NLRP3—Nod-like receptor pyrin-containing 3 subunit

Diabetes significantly increased the level of the NLRP3 subunit (*p* < 0.05), while the escalating effect of LPS (1 μg/ml, 24 h) was observed only in the control cultures (*p* < 0.05) (Fig. 6a). No effects of metformin (3 μM, 24 h) or glyburide (1 μM, 24 h) were found in the control group, but analysis showed that, in the diabetic group, glyburide (1 μM, 24 h) significantly decreased the level of the NLRP3 subunit (*p* < 0.05). Moreover, the LPS-evoked increase in the NLRP3 subunit protein level in the control cultures was attenuated by glyburide (1 μM, 24 h; *p* < 0.05), while in the diabetic cultures, it was diminished by metformin pretreatment (3 μM, 24 h; *p* < 0.05).

2. **ASC**

#### Protein Level in the Homogenates of Hippocampal Organotypic Cultures

None of the factors affected the level of the ASC protein in the hippocampal cultures in the examined groups (Fig. 6b).

3. **Caspase-1**

#### Protein Level in the Homogenates of Hippocampal Organotypic Cultures

In the diabetes group, we observed a tendency towards an increase in the caspase-1 level, while glyburide treatment (1 μM, 24 h) diminished this factor in this group (*p* < 0.05) (Fig. 6c). Both LPS stimulation (1 μg/ml, 24 h) and metformin administration (3 μM, 24 h) in the control cultures enhanced caspase-1 protein levels (*p* < 0.05); in the diabetic cultures, these effects were not observed.

## Discussion

Our study advances the two-hit hypothesis, which indicates that maternal diabetes increases hippocampal sensitivity to LPS-triggered inflammatory responses in offspring brain. In addition, we demonstrate the strong inhibitory properties of metformin and glyburide on immunoactivation induced by maternal diabetes in the hippocampus of offspring. In our study, the effects of glyburide were more pronounced.

The present study showed that hippocampi derived from the offspring of diabetic mothers are characterized by an increase in cell death and a higher susceptibility to damage caused by a second stressor, specifically bacterial endotoxin treatment. This effect may be explained by the hypothesis that hyperglycemia in utero may induce oxidative stress (the literature confirms that STZ leads to the overproduction of superoxides, peroxynitrates, hydroxyl radicals, and hydrogen peroxide, which are responsible for DNA damage and fragmentation, characterizing the genotoxic potential of STZ; Vikram et al. 2007), apoptosis in target tissues and the alteration of the expression of genes involved in the proliferation and differentiation of brain cells, especially the downregulation of anti-apoptotic Bcl-2 and the upregulation of pro-apoptotic Bax (Hami et al. 2013, 2015; Haghir et al. 2017). Direct STZ cytotoxicity has also been demonstrated in hypothalamic and hippocampal neurons in STZ-treated animals and is evaluated by a decrease in the number of neurons and morphological changes in these animals (Bathina et al. 2017). Diabetes-induced apoptosis in hippocampal neurons may be a key mechanism explaining memory and learning defects observed in the offspring brain (Razi et al. 2015; Tan et al. 2015). Maternal diabetes may also sensitize offspring brain cells to other negative factors. Our study revealed that stimulation with LPS causes an increase in LDH release and NO levels in the medium, while metformin and glyburide show protective effects against LPS-dependent adverse changes. These results are in line with previous findings (El-Mir et al. 2008; Chung et al. 2015; Chen et al. 2016; Zhou et al. 2016), which reported that metformin treatment may cause a wide range of beneficial effects on brain functions after injury. For example, in cultured brain cells, metformin prevents neurodegeneration induced by advanced glycation products in human neuronal stem cells (Chung et al. 2015), glutamate-induced toxicity in cultured cerebellar granule neurons (Zhou et al. 2016), and neurotoxicity caused by etoposide (El-Mir et al. 2008) and amyloid-β (Chen et al. 2016) in hippocampal neurons in vitro. Similarly, glyburide has been shown to have pleiotropic neuroprotective effects. This antidiabetic agent is able to inhibit necrotic cell death, reduce posttraumatic brain edema, and decrease contusion volume following open head injury in rats and possesses long-term protective effects on the rat hippocampus after traumatic brain injury (Kurland et al. 2013; Xiong et al. 2015).

The important finding from the present study is the observation that maternal diabetes increases the levels of pro-inflammatory cytokines, including IL-18 and IL-6, in the homogenates of organotypic hippocampal cultures. The data showed that IL-6 is a multifunctional cytokine involved in the inflammatory response and is correlated with insulin-resistant states. Moreover, increased IL-6 levels can predict disease progression in patients with diabetes (Gomes 2017). Studies in Wistar rats have shown that diabetes in adult animals leads to IL-6 overexpression (Feng et al. 2018) in the hippocampus and, as our study indicates, maternal diabetes may exert a similar effect in the brains of offspring. Diabetes did not cause changes in the levels of the other cytokines tested in homogenates, but the most important assessment was the effect of diabetes on secretion of these cytokines under the influence of a second stimulus, LPS. In fact, we have showed that the LPS-stimulated secretion of IL-1β and IL-6 is higher in cultures obtained from the hippocampi of the offspring of diabetic dams. Data have shown that metformin possesses anti-inflammatory properties, manifested by the inhibition of microglial activation and the downregulation of pro-inflammatory cytokine production (TNF-α, IL-1β, and IL-6) in a rat traumatic brain injury (TBI) model (Tao et al. 2018). Moreover, the protective effect of metformin against ischemic changes via the suppression of the NF-κB-mediated inflammatory pathway has been demonstrated (Karimipour et al. 2018). Therefore, the suppressive impact of metformin on LPS-induced IL-1β and IL-6 secretion observed in the present study seems to be partially in line with those observations. To date, only a few studies have demonstrated the anti-inflammatory properties of glyburide in the brain. For example, glyburide attenuates ischemia-reperfusion injury by modulating oxidative stress and inflammatory factor secretion (NO, TNF-α) into the medium in the rat hippocampus (Abdallah et al. 2011). To our knowledge, no studies addressing the impact of glyburide on LPS-stimulated pro-inflammatory cytokine secretion in the brain exist, so we propose that the present data are the first showing that this drug may act as an anti-inflammatory agent and normalize the LPS-evoked secretion of IL-6, TNF-α, and IL-1β into the medium in both control and diabetes hippocampal cultures.

A major challenge in understanding inflammatory processes in the brain is determining the role of the main pro-inflammatory cytokine IL-1β because it may induce the secretion of other pro-inflammatory cytokines and chemokines in addition to inducing its own secretion and initiate substantial inflammation (Basu et al. 2002). Although the basal level of IL-1β in the brain is low, it is strongly induced immediately after injury or upon stimulation by inflammatory agents. In our study, we observed the increased secretion of IL-1β in diabetic and control hippocampi after LPS challenge (in diabetic cultures, IL-1β secretion after LPS was even higher than that in controls). We found that both metformin and glyburide inhibit the secretion of IL-1β from control and diabetic cultures stimulated with LPS and that the effect of metformin is stronger in diabetic hippocampi than in controls. Another research group reported similar effects in an in vivo study on diabetes; in the hippocampi of STZ mice, the expression of IL-1β is greater than that in the controls, and metformin reduces this effect (Oliveira et al. 2016). IL-1β has been recognized as important in the progression of insulin resistance and diabetes (Maedler et al. 2009), and the severe action of metformin can be explained by its ability to counteract insulin resistance mediated by IL-1β. Interestingly and unexpectedly for us, we observed that metformin alone causes an increase in IL-1β protein levels in control hippocampi (versus controls treated with vehicle), and this effect was not observed in the diabetic group. Likewise, other findings have proven that metformin alone has no effect on the production of pro-inflammatory cytokines, except for that of IL-1β (Łabuzek et al. 2010), but this observation certainly requires further study. Additionally, the effects of maternal diabetes and/or LPS on cytokine levels measured in the culture medium and homogenates are not always parallel, presumably because cytokines are mainly released through the classical form of exocytosis. Additional harmful factors do not affect cytokine storage but influence the secretion process. In the presence of an inflammatory agent, cytokines are produced and directly secreted; they are not stored. After LPS challenge in slices, we observed an increase in the gene expression and secretion of the investigated cytokines (IL-1β, IL-18, IL-6, and TNF-α). This lack of parallelism in the levels of individual cytokines in the medium and homogenates may also result from differences in their release or degradation. For example, IL-1β, unlike most cytokines, does not have a secretory signal sequence and is not secreted by the classical endoplasmic reticulum-Golgi pathway. The exact mechanism of IL-1β secretion from the cytosol to the extracellular space is not known, and depending on the cell types and inflammatory conditions, several different pathways have been proposed (e.g., through the externalization of the cytokine directly at the plasma membrane, via vesicles, via the autophagy process, through microvesicles shed from the plasma membrane, and via exosomes) (Piccioli and Rubartelli 2013; Iula et al. 2018). In addition, in the case of IL-18 and IL-6, the increased secretion of these cytokines under the influence of LPS in the group with diabetes may be the result of the increased production of these factors in the slices under basal conditions.

Data have indicated that NLRP3 inflammasome platform activation triggers the proteolytic cleavage of pro-caspase-1 into active caspase-1 and leads to the maturation of IL-1β from its precursor form (Kang et al. 2016; Swaroop et al. 2018). It was shown that the activation of the NLRP3 inflammasome is precisely regulated; its activation involves priming induced by the Toll-like receptor (TLR) and nuclear factor (NF-κB) pathways and then assembly of the NLRP3 inflammasome, caspase-1 activation, and IL-1β secretion (Dowling and Neill 2012). The first step may be facilitated through the activation of NOD-like or Toll-like receptors. Among these receptors, TLR4 acts as a major LPS signaling receptor leading to the inflammatory response. In our study, we demonstrated that TLR-4 levels are upregulated in diabetic organotypic cultures. Furthermore, additional LPS stimulation leads to TLR4 upregulation in control and diabetic hippocampal cultures. Studies have shown that the consumption of high levels of dietary fats leads to the activation of metabolic inflammation in the hypothalamus through TLR4, and the inhibition of TLR4 significantly reduces hypothalamic inflammation in the hypothalami of rodents fed a high-fat diet (Milanski et al. 2009; Velloso et al. 2015). Investigations have discerned the role of TLR4 in peripheral tissues; in type 1 diabetes patients, even small amounts of glucose induce oxidative and inflammatory stress, which is reflected by, among other effects, TLR4 activation. In addition, in these patients, insulin treatment shows anti-inflammatory effects, including the suppression of TLR4 (Dandona et al. 2013). Additionally, other groups have provided evidence for the involvement of TLR4 in the pathogenesis of obesity and insulin resistance (Kang et al. 2016).

Because the activation of TLR4 may lead to the stimulation of the transcription factor NF-κB and, as a consequence, to the intensification of inflammatory cytokine production, in our study, we investigated the impact of diabetes on the phosphorylation of the p65 NF-κB subunit. Under basal conditions, NF-κB is maintained in the cytoplasm as a non-activated molecule. In response to stimuli, the activated NF-κB subunit p65 translocates to the nucleus and promotes the specific gene transcription of cytokines (e.g., TNF-α, IL-1β, and IL-6). Generally, this signaling pathway leads to inflammation and neuronal apoptosis (Shao et al. 2016). We observed the upregulation of the phospho-p65/p65 ratio after LPS stimulation. This effect was still observed in groups pretreated with metformin, but interestingly not after glyburide pretreatment, which suggests the inhibitory impact of this drug on NF-κB phosphorylation and probably a reduction in pro-inflammatory factor secretion. Some data have demonstrated that TLR4-induced NF-κB activation is involved in the regulation of NLRP3 transcription through binding to the NF-κB binding sites in the NLRP3 promoter (Qiao et al. 2012). In fact, once primed, the subsequent activation of the NLRP3 inflammasome, referred to as the second signal, results in the oligomerization of NLRP3 and the subsequent assembly of NLRP3, ASC, and pro-caspase-1 into a platform (Lamkanfi and Kanneganti 2010). In the present study, maternal diabetes was able to regulate the second signal of the NLRP3 inflammasome activation pathway. In fact, we observed that maternal diabetes causes the activation of NLRP3 inflammasome signaling by increasing the level of the NLRP3 protein subunit, indicating a tendency to enhance the protein levels of caspase-1 and ASC. Interestingly, we found that glyburide, as a NLRP3 inflammasome inhibitor, diminishes the level of NLRP3 protein and caspase-1 subunits, but only in diabetic cultures. Recent data have indicated that NOD-like receptors (NLRs) are not only essential components of the immune system but may also have an important role as regulators of the glucose and insulin balance (Vandanmagsar et al. 2011). This association between metabolic stress and inflammation suggests a link between inflammasome activation, especially the activation of NLRP3, and insulin resistance (Stienstra et al., 2011). It seems that the activation of the NLRP3 inflammasome is an important mechanism that induces metabolic-related inflammation and low sensitivity to insulin. Our study showed that maternal diabetes can lead to NLRP3 inflammasome activation in the offspring hippocampus, which may suggest a relationship between the dysregulation of the maternal insulin signaling pathway and inflammatory changes in offspring brains. Only somewhat related results have been shown in immortalized mouse HT22 hippocampal cells, in which high glucose levels increase NLRP3 markers and activation compared to those induced by normal and low glucose levels (Ward and Ergul 2016). However, many reports refer to the activation of the NLRP3 inflammasome in peripheral tissues in diabetes. High glucose levels are associated with the activation of the NLRP3 inflammasome. Elevated NLRP3, ASC, IL-1β gene expression, and protein levels are observed in macrophages obtained from untreated type 2 diabetic patients. Furthermore, in the myeloid cells of type 2 diabetes, caspase-1 activation is observed. This effect leads to the upregulation of IL-1β and IL-18 levels (after exposure to metabolic danger signals). Importantly, the knockdown of NLRP3 and ASC subunits in the myeloid cells of type 2 diabetic patients prevents the ability of metabolic signals to induce IL-1β and IL-18 secretion (Lee et al. 2013).

In conclusion, the current results clearly demonstrated that maternal diabetes increases the mortality of hippocampal cells and enhances their susceptibility to damage and inflammation caused by a second stressor, lipopolysaccharide. The investigated antidiabetic drugs with anti-inflammatory properties (metformin and glyburide) have a beneficial impact on the observed dysregulations. The application of the NLRP3 inflammasome inhibitor glyburide appears to be the most promising and has particular therapeutic value. We suggest the potential utility of this drug as a new therapeutic strategy in brain disorders in which inflammation is observed. The search for new therapeutic opportunities to counteract immunometabolic brain disorders linked to resistance to conventional treatments is a short-term necessity.

## List of Abbreviations

ANOVA: Analysis of variance
ASC: Apoptosis-associated speck-like protein containing a caspase recruitment domain
BGC: Blood glucose concentration
casp1: Caspase-1
Ctrl: Control
DMEM: Dulbecco’s modified Eagle medium
ELISA: Enzyme-linked immunosorbent assay
FACS: Fluorescence-activated cell sorting
GLI: Glyburide
HBSS: Hank’s balanced salt solution
HEPES: 4-(2-hydroxyethyl)-1-piperazineethanesulfonic acid
HS: Horse serum
IL: Interleukin
LDH: Lactate dehydrogenase
LPS: Lipopolysaccharide
MET: Metformin
NF-кB: Nuclear factor kappa-light-chain-enhancer of activated B cells
NLRP3: Nod-like receptor pyrin-containing 3 inflammasome
NO: Nitric oxide
OHC: Organotypic hippocampal cultures
PBS: Phosphate-buffered saline
PE: Phycoerythrin
PI: Propidium iodide
PVDF: Polyvinylidene difluoride
RIPA: Radioimmunoprecipitation assay
SEM: Standard error of the mean
STZ: Streptozotocin
TBS: Tris-buffered saline
TLR: Toll-like receptor
TMB: 3,3′,5,5′-tetramethylbenzidine
TNF-α: Tumor necrosis factor α
